# Social Transformation in Environmental Awareness: The Role of Hospital Employees in the Transformation Process—Results of a Survey in Germany

**DOI:** 10.3390/bs16040622

**Published:** 2026-04-21

**Authors:** Berit Schoppen, Katja Falta, Thomas Ostermann

**Affiliations:** 1 MedEcon Ruhr GmbH, 44801 Bochum, Germany; 2 Department of Psychology and Psychotherapy, Witten/Herdecke University, 58455 Witten, Germany; katja.falta@uni-wh.de (K.F.); thomas.ostermann@uni-wh.de (T.O.)

**Keywords:** green hospitals, environmental awareness, hospital employees, attitudes, behavior, path analysis

## Abstract

Against the backdrop of increasing challenges posed by ongoing climate change, pollution and its associated implications and environmental and climate protection measures have become the focus of politics and society in recent years. But adaptation and mitigation require changes in behavior among the population. This means that a process of social transformation must take place. Based on the thesis that people working in healthcare facilities can make a relevant contribution to the social transformation of environmental awareness, this study examines the general and workplace-related environmental awareness of hospital employees. Risk awareness, measure effectiveness, transformation costs and the subjective social norm factors were included, which eventually influence this awareness. Therefore, a mixed study design consisting of an original study and a systematic replication study was used. Data were analyzed using structural equation modeling (path analysis) to identify key predictors of environmental awareness and behavior. The results show that hospital employees generally have a high level of environmental awareness compared to the general population in Germany. This is also evident in their awareness of workplace-related measures. However, influencing factors such as transformation costs in particular mean that measures, even if they are considered effective, are only implemented to a limited extent by hospital employees. With regard to the question of whether hospital employees can play a key role in the social transformation process, this study does not provide sufficient evidence to answer this question.

## 1. Introduction

Against the backdrop of increasing challenges posed by ongoing climate change and its associated implications, such as hot summers, forest dieback, and high incidence of forest fires, environmental and climate protection measures have become the focus of politics and society in recent years. The same applies to the effects of environmental pollution, such as littering in the oceans and air pollution (Brasseur & Jacob, 2017). Health-related consequences manifest themselves in latent (e.g., heat, changes in epidemiological events, water or food shortages), but also in acute threats (e.g., forest fires or floods) (Romanello et al., 2021; Tennison et al., 2021; United Nations Framework Convention on Climate Change, 2019).

Two different social strategies incorporating public and private ambitions can be pursued in response to environmental and climate change: adaptation and mitigation. According to the pioneering definitions by the Intergovernmental Panel on Climate Change (IPCC), adaptation involves adjustments in natural or human systems in response to actual or expected climatic stimuli or their effects, which moderate harm or exploit beneficial opportunities (Klein et al., 2007; Watson, 2001). Adaptation always represents a response to changing climatic conditions. This is regardless of the fact that these changed conditions can be anticipated in advance, allowing for a proactive response. The primary objective is to ensure that systems are sufficiently resilient, which includes measures such as planting heat-resistant forests, creating water reservoirs, but also disaster control measures and heat protection plans, or installing early-warning systems (Watson, 2001).

Given that climate change is ongoing, there is also the question of how the remaining scope for action can be used to mitigate the effects of climate change and prevent ecological tipping points from being reached. Mitigation, as defined in the framework of the United Nations Framework Convention on Climate Change (UNFCCC), refers to anthropogenic interventions to reduce the sources or enhance the sinks of greenhouse gases (United Nations Framework Convention on Climate Change, 2019; Klein et al., 2007; Watson, 2001; Intergovernmental Panel on Climate Change, 2007). Ecological tipping points refer to critical thresholds beyond which developments become irreversible, leading to chain reactions across subsystems—such as the melting of polar ice sheets, permafrost degradation, or the collapse of the Amazon rainforest (Rahmstorf & Schellnhuber, 2019).

Early assessments by the IPCC, specifically the Third Assessment Report Intergovernmental Panel on Climate Change (2001), already emphasized that mitigation requires a broad portfolio of technologies, but also significant shifts in social and individual behavior to achieve stabilization goals. With the establishment of a framework agreement on stabilizing greenhouse gas concentrations by the UNFCCC and the adoption of the Kyoto Protocol (United Nations, 1997), attempts were made worldwide to address this. However, as underscored in the Fourth Assessment Report (Intergovernmental Panel on Climate Change, 2007), it must be assumed that these commitments and the pace of their implementation are insufficient to effectively curb the advance of climate change (Intergovernmental Panel on Climate Change, 2007).

In any case, both areas—adaptation and mitigation—require significant changes in behavior among the population, i.e., a process of social transformation must take place. While adaptation is often driven by necessity, mitigation requires a high degree of intrinsic motivation on the part of the individuals involved, as it addresses a global collective action problem (Klein et al., 2007; Intergovernmental Panel on Climate Change, 2007). This study examines the social transformation process primarily from the perspective of mitigation, aiming to reduce environmentally harmful emissions through both technological innovation and profound behavioral changes in energy and resource consumption (Otto et al., 2020). Particularly, the latter requires society to be willing to change its behavior in all areas of life and work.

### 1.1. Options for Hospital Staff to Influence Adaptation and Mitigation

Healthcare facilities and their employees have the opportunity to contribute to improving the environmental balance of the healthcare sector through various approaches. On the one hand, they can use their reputation among the population to influence the transformation process in society as a whole. On the other hand, they have their own scope for action to reduce environmentally harmful consumables and emissions. Measures such as waste and emission avoidance can be implemented in the short term and without major investment costs. In addition, it is important to address the question of how unavoidable resource consumption can be powered by renewable energy sources and how unavoidable waste can be recycled (Dickhoff et al., 2021). The potential for relevant effects is great because the healthcare sector is responsible for 4.4% of global CO_2_ emissions; in Germany, this figure is as high as 5.2% (Karliner et al., 2019; Romanello et al., 2025).

Both approaches, alone or in combination, can have an overall effect on achieving social tipping points. This term describes the points in the social transformation process at which a change in the environmental awareness of society as a whole occurs. It is assumed that a critical mass must be reached before society as a whole will rethink its position and consistently align its behavior with an effective mitigation process. The term is borrowed from the field of natural sciences (Milkoreit et al., 2018). In particular, the health professions in the German professional community are considered to have great potential to influence the achievement of such social tipping points. It is assumed that linking environmental pollution to health risks can be the decisive factor in bringing about a change in society’s thinking, thereby significantly accelerating the achievement of the social tipping points described (Dobson et al., 2021; Mezger et al., 2021, Wild, 2025). Table 1 provides examples of measures from the healthcare sector that contribute to this goal.

With regard to the anticipated influence of healthcare workers, this paper will focus on hospital employees in Germany. This paper also focuses primarily on the area of behavioral adjustment in the transformation process. The transformation process considered refers to a change in values that engenders the necessary environmental awareness, which in turn is defined as a prerequisite for consistently environmentally friendly behavior.

### 1.2. Potential and Restrictions for the Role of Hospital Employees as Promoters of the Transformation Process

Discussions among experts suggest that healthcare professionals themselves act as a homogeneous group with a uniformly high level of climate, environmental, and health awareness, already implement measures to a large extent themselves, and can therefore act as so-called environmental activists (Kuckartz et al., 2007) and as multipliers for the goals set in the transformation process. However, it remains questionable whether hospital employees inevitably act in an environmentally conscious manner solely due to their knowledge of the links between environmental factors and health.

It can be assumed that the mechanisms that lead to certain human actions are not exclusively shaped by attitudes. Social psychological theories such as the Theory of Reasoned Action (Ajzen, 1980; Fishbein & Ajzen, 1975), the Theory of Planned Behavior (Ajzen & Madden, 1986; Ajzen, 1991), or the Social Cognitive Theory (Bandura, 1986, 1997) are based on complex mechanisms in which a wide variety of factors influence the formation of behavioral intentions and subsequent behavior. Attitudes, subjective norms, and perceived behavioral control interact in a complex way before any intention is formed. Other factors, such as actual behavioral control, functional optimism, or cognitive dissonance, can also influence whether intentions actually lead to a certain behavior—in this case, environmental or climate-related behavior—even after the intention has been formed. In addition, there are economic incentive factors that are expressed in opportunity costs or cost–benefit assessments (also referred to as behavioral costs) (Diekmann & Preisendörfer, 1992). As early as the late 1980s and early 1990s, initial studies demonstrated a clear link between environmental awareness and environmental behavior (Diekmann & Preisendörfer, 1991; Hines et al., 1987; Langheine & Lehmann, 1986). However, this correlation is so weak that the intended behavioral adjustment often depends on “optimal” conditions, or rather, the behavioral adjustment must not be counteracted by overly strong counter-inducing factors (Preisendörfer, 1999). Consequently, it became apparent that environmental awareness alone cannot be a sufficient explanatory factor for behavioral adaptation (Diekmann & Preisendörfer, 1992). Rather, there are such strong divergences between environmentally related intentions and the associated behavior that environmental awareness (i.e., attitudes) serves as only a weak predictor of environmentally friendly behavior.

On the one hand, hospital employees, and, in particular, healthcare professionals, can be assumed to have a particularly high-risk awareness posed by environmental and climatic influences. This would support the thesis that this group of people has an intrinsic motivation to behave in an environmentally conscious manner. On the other hand, hospitals and their staff are subject to special conditions and requirements, such as hygiene regulations. Employees also have only limited influence on supply chains or can only oversee them in a rudimentary way. At the same time, hospitals in Germany are currently subject to strong economic constraints, which are reflected, among other things, in an investment backlog that has built up over decades (Augurzky & Kolodziej, 2018; Augurzky et al., 2025; Graalmann et al., 2021), which also coincides with a period in which a comprehensive, nationwide hospital reform is being undertaken in Germany (BGBl, 2024; Weller, 2024). Further environmental adaptation measures in processes and structures would thus lead to transformation costs (Wittenberg et al., 2021), the amount and scope of which may be assessed differently by the actors involved.

In addition, advocating environmental and climate protection measures is not always appropriate. In this respect, it is not only relevant whether hospital employees themselves have a high level of environmental awareness. It is also relevant which, as subjectively perceived social norms, are conveyed and practiced in this regard within the institution. This is because adapting behavior, even with a high level of personal environmental awareness, against possible resistance would also require a low degree of self-monitoring (Collani & Stürmer, 2009; Snyder, 1974).

Finally, assessing the effectiveness of the various environmental *measures* that can be implemented in hospitals may represent a further obstacle to the implementation of these measures. If measures and proposed actions are not considered effective in terms of environmental protection, or if it is assumed that these measures will only have a minor impact, this can affect the willingness to implement the measures and adapt behavior accordingly (Esser, 1999; Lindenberg, 1985).

### 1.3. Subjects of Investigation in This Study

Based on the thesis that people working in healthcare facilities can make a relevant contribution to achieving social tipping points through their work in healthcare, this study examines both the environmental awareness of hospital employees and their environmental behavior in order to derive potential for the social transfer process.

**Figure 1:**
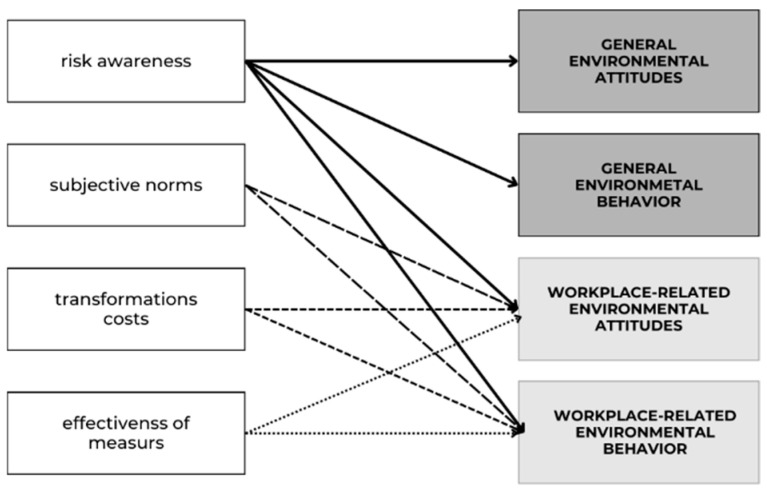
Potential factors influencing environmental attitudes and behavior. Different arrow types and shading of the boxes are used to improve visualization and have no specific meaning.

Even if hospital employees are highly environmentally conscious—which is only an assumption at this point and still needs to be proved— and even if this awareness would be translated into corresponding behavior, further constellations are needed with regard to risk awareness, assumptions about *transformation costs* and the effectiveness of measures, as well as subjective social norms, so that environmentally conscious hospital employees actually adapt their own behavior and can also act as multipliers in society (see Figure 1).

The primary objective of this study was to evaluate the level of environmental awareness among hospital employees and to determine how this awareness compares to that of the general population in Germany. Beyond general awareness, the research investigated whether hospital staff possess a more extensive knowledge base regarding the global impacts of environmental pollution and climate change, and whether such knowledge directly correlates with their environmental attitudes.

Furthermore, the study sought to identify additional factors influencing environmental awareness within the clinical setting. A particular focus was placed on the role of workplace-related measures; while employees may recognize the effectiveness of such interventions, their implementation is often hindered by specific barriers. Drawing on low-cost theory (Ajzen, 1991), this research examined the assumption that transformation costs significantly impede the adoption of pro-environmental behaviors. To rigorously test these relationships and address the underlying determinants of environmental awareness, a path analysis was conducted, allowing for a comprehensive evaluation of the direct and indirect influences of knowledge, attitudes, and perceived costs.

## 2. Materials and Methods

### 2.1. Study Design

A mixed-method design was employed involving an original study and a systematic replication to evaluate the research questions through two different instruments. The original study was conducted in the form of a vignette study based on the factorial survey method (Beck & Opp, 2001; Reineck et al., 2017), which was used to survey environmental attitudes and behavior with explicit reference to the hospital working environment and, within that context, to specific environmental measures in the workplace. In addition, the respondents’ assessment of the effectiveness of the measures and the anticipated transformation costs of these measures were also surveyed.

The replicating component was based on the series of environmental awareness studies conducted every two years by the German Environment Agency (UBA). As part of this series, a further development for surveying environmental attitudes (environmental affect, environmental cognition, and intentional environmental behavior) was developed in 2018 (Geiger, 2020). The primary data collected from the target group of hospital employees was compared with the corresponding results of the representative survey conducted by the UBA in 2018 and 2022 for classification purposes. Risk awareness was also surveyed using a battery of items from the aforementioned study, which was used by the UBA in 2022 and whose results were compared with the results of the present survey. The questionnaire was conducted as an online survey during the third and fourth quarters of 2024.

### 2.2. Participants and Sample Size

Hospital and rehabilitation clinic employees were eligible to participate in the study regardless of their age or occupational group. The inclusion criteria were current employment in a hospital or rehabilitation clinic, or a previous employment that ended no longer than eight years ago.

Regarding field access, the study relied primarily on the authors’ personal contacts, who maintain an extensive professional network within the hospital landscape, especially in the Ruhr area. These contacts were personally invited to participate in the survey. Members of all occupational groups in the hospital were eligible. Participation was facilitated via an email link available from August to December 2024. Some of the hospital employees who were personally approached also acted as multipliers by forwarding the link to their colleagues. This snowball sampling method enabled the recruitment of participants from across Germany, extending beyond the initial region.

The sample size required to test the research question was calculated based on a significance level of *α* = 0.05, an effect size of *d* = 0.5, and a power of 80%. Accordingly, a minimum of 128 fully completed data sets was required to address the primary research question.

Prior to the start of the survey, all participants were informed about the topic, the voluntary nature of their participation, the confidential handling and pseudonymization of data, and the expected duration of the survey, and they had to confirm that they were providing informed consent. Ethical approval was obtained from the Ethical Committee of Witten/Herdecke University (ID: S-158/2024; approved on 17 June 2024).

### 2.3. Instruments and Methodological Procedure

#### 2.3.1. Replication Instruments

General Environmental Attitudes: Environmental attitudes were assessed using an item battery derived from the environmental awareness scales developed for the German Environment Agency (UBA) representative surveys (Geiger, 2020; Stieß et al., 2020). This longitudinal survey is conducted biannually by the UBA. In 2018, a validated 23-item battery was introduced, comprising three subscales: environmental affect (impact), environmental cognition, and intentional environmental behavior (Stieß et al., 2020; Rubik et al., 2021). To contextualize the hospital employees’ scores, their results were compared with the mean scores from the 2018 and 2022 UBA representative surveys (Stieß et al., 2020; Rubik et al., 2021). High scores represent a high level of environmental awareness, which is considered a key prerequisite for the willingness to implement environmental measures (Geiger, 2020; Stieß et al., 2020). In the present study, the workplace-related environmental attitudes (WEA) scale demonstrated acceptable internal consistency (*α* = 0.83).

While the 2018 UBA survey employed a four-point Likert scale, the 2022 representative survey transitioned to a six-point Likert scale. In their final report on the representative survey, the authors provide coding instructions to ensure comparability of the results of 2022 and 2018. These coding instructions were applied here. For the analysis of the item batteries on general environmental attitudes (Geiger, 2020; Stieß et al., 2020), the stored item values therefore first had to be coded. For this purpose, the values of the item batteries on environmental impact and environmental cognition, which were surveyed using a four-point Likert scale (“strongly disagree”, “tend to disagree”, “tend to agree”, “strongly agree”) and which were previously assigned values of 0–3, were adapted to the value labels of the six-point Likert scale (0 = never, 5 = always) used in the UBA representative survey 2018 and 2022. Therefore, the previous values had to be rescaled. The value labels of the dichotomous question on environmental behavior (0 = no; 1 = yes), on the other hand, were multiplied by a factor of 5. This coding was necessary to ensure comparability with the results of the corresponding item batteries of the representative surveys of 2018 and 2022 (Rubik et al., 2021). Negatively phrased items (e.g., “Other things are more important for a good life than the environment”) were reverse-coded so that higher scores consistently reflect a higher level of environmental awareness.

General Environmental Behavior: In addition to attitudes, general environmental behavior was also assessed to address the study’s primary objective. The General Ecological Behavior Scale (GEB 50) was employed to evaluate whether hospital employees tended toward environmentally friendly or harmful behaviors on average. The workplace-related environmental behavior (WEB) subscale demonstrated acceptable internal consistency (*α* = 0.73). For its evaluation, the responses for items 1–32, which were collected via a Likert scale, were dichotomized in accordance with the procedural documentation (Grothmann et al., 2023; Kaiser, 2020).

Risk Awareness: Risk awareness was assessed using a second set of items (i.e., perceived damage and personal impact), which also derived from the 2022 UBA representative survey conducted (Umweltbundesamt, 2023), which focused on perceived environmental risks and the potential threats to personal health. These results were also compared with the reference data of the 2022 UBA representative survey (Grothmann et al., 2023).

Methodological Adjustments for Risk Awareness Scales: In the original 2022 UBA survey, the item battery regarding anticipated health damage (F 3.1) employed a five-point Likert scale, while the battery on perceived threats from environmental influences (F 4.2) used a four-point Likert scale. However, in the present study, these two scales were inadvertently swapped during the survey administration. Consequently, a direct comparison with the 2022 UBA representative survey data was not immediately feasible. To ensure comparability, nonetheless, the data were transformed using the rescaling procedure described in Appendix A (see Table A1 and Table A2). This rescaling procedure did not affect the construct validity of the questionnaire. The two scales concerned, “harm” and “threat,” showed a high internal consistency measured by Cronbach’s alpha of 0.83 and 0.88, respectively.

Furthermore, the effectiveness measures (EM) scale exhibited good reliability (*α* = 0.81). The scale for transformation costs (TC) reached an internal consistency of *α* = 0.67. While slightly below the conventional 0.70 threshold, it was deemed acceptable for exploratory analysis given the specific context of hospital environments.

Following this adjustment, statistical analyses were conducted, including a demographic analysis by gender, age, and occupational group. Furthermore, correlation analyses were performed to examine the relationships between risk awareness, environmental attitudes, and environmental behavior.

#### 2.3.2. Original Study Instruments

Workplace-related Environmental Attitudes and Behavior, Effectiveness and Transformation Costs: The elicitation of workplace-related environmental attitudes was part of a factorial survey. For this purpose, an overview of imaginable implementation measures for environmental and climate protection in hospitals (see Table 2), as well as their implementation requirements and potential implications for the various occupational groups in the facilities, based on a literature review, was obtained.

These possible measures were then operationalized for the factorial survey (Table 3).

Respondents were asked to imagine such situations and answer whether they considered these measures to be appropriate and how they assessed the potential for implementing these measures in hospitals. The survey focused on (a) workplace-related environmental attitudes, (b) the effectiveness of measures, (c) the transformation costs, and (d) the willingness to implement these imaginable measures.

Data for the factorial survey were also collected using a four-point Likert scale and were coded in the same way as the item batteries on environmental impact and environmental cognition (“completely disagree”, “tend to disagree”, “tend to agree”, “completely agree”). Through this approach, the results of the exploratory survey of the factorial survey could be classified and interpreted by comparing them with the results of the UBA’s item batteries. Subsequently, the workplace-related environmental attitudes and the general environmental attitudes were analyzed using Pearson’s bivariate correlation analysis to identify potential relationships.

Subjective Social Norms: According to the theory of subjective norms, it is assumed that a perceived environmental organizational policy (i.e., normative conviction) can have a positive influence on the environmental and climate attitudes of the respondents (Ajzen, 1980). A self-designed questionnaire was used to survey normative beliefs, asking about various potential green hospital measures in terms of the company’s expectations for their implementation. The content of the questionnaire was also based on the examples of measures listed, so that the subjective norms were surveyed in the same way as the examples of measures in the factorial survey. Assuming that, for the purposes of the study, there is a positive correlation between workplace-related environmental attitudes, workplace-related environmental behavior, and the willingness to implement measures in the workplace, the results of these observations were correlated with the results of the subjective norms.

To ensure a meaningful evaluation, the subjective norms response values were first coded (Table A3). This was followed by a descriptive analysis of the frequency distributions for the subjective norms of the overall scale (average score values achieved in the seven individual items per respondent) and along the individual questions. To determine the correlations between environmental attitudes and behavior, a bivariate Pearson correlation analysis was conducted.

### 2.4. Statistical Analysis

Statistical analyses were conducted using IBM SPSS Statistics (Version 29; IBM Corp., 2022, Armonk, NY, USA), R Core Team (2025, Version 4.5.3; R Foundation for Statistical Computing, Vienna, Austria), and Mplus (Version 8.11; Muthén & Muthén, 2017, Los Angeles, CA, USA). Descriptive statistics and correlation analyses were performed in SPSS and R, while the path analysis was carried out via the R package MplusAutomation (Version 1.2; Hallquist & Wiley, 2018).

According to Barbeau et al. (2019) we specified a saturated path model to estimate the effects of psychological predictors (efficacy, threat, harm, social subjective norms, and transformation costs) and control variables (age, gender, and occupational groups) on four endogenous variables: general environmental attitude (GEA), general environmental behavior (GEB), workplace-related environmental attitude (WEA), and workplace-related environmental behavior (own willingness, WEB_O).

Model parameters were estimated using Maximum Likelihood (ML). To evaluate the magnitude of effects, the proportion of explained variance (*R*^2^) and standardized path coefficients (*β*) are reported. Significance level was set at *α* = 0.05.

## 3. Results

### 3.1. Sample Description

A total of 163 people participated in the study, 130 of whom fully completed the questionnaire. In accordance with the study protocol, data analysis was restricted exclusively to participants with complete datasets.

Of the 130 participants, 94 (72.3%) were female, and 36 (27.7%) were male. No other genders were specified. The age ranged from 24 to 65 years, with a median age of 42 years. Categorized by ten-year increments, the largest group consisted of participants aged 31–40 years (*n* = 43), followed by those aged 41–50 years (*n* = 38) (Table 4).

Regarding the occupational background of the total sample, medical services constituted the largest professional group, representing 42.3% (*n* = 55) of all participants. The remaining sample was distributed among administrative staff (17.7%, *n* = 23), nursing staff (16.2%, *n* = 21), and a collective group of other professions (23.9%, *n* = 31), which comprised individuals from IT services, kitchen, management, and department heads. The occupational categories building services, cleaning services, and pharmacy were not represented at all.

A gender-specific analysis across these categories showed that the medical services had the highest representation of both genders, though it was predominantly female-led (*n* = 35 female, *n* = 20 male). The nursing staff and administrative staff were also characterized by a clear female majority, 81.0% and 65.2%, respectively. Notably, the “Others” category showed the most pronounced gender disparity, with 27 out of 31 participants identifying as female, i.e., 87.1%.

### 3.2. General Environmental Attitudes

In comparison with the results of the UBA representative surveys 2018 and 2022, the hospital employees in the sample show a highly significant upward deviation in the environmental awareness scores. This difference is present for overall environmental awareness and in two of the three subscales: environmental effect (EE) and intentional environmental behavior (EB) (Table 5).

An examination of the different age groups reveals that participants aged 61–70 years exhibit higher values in all three areas of environmental awareness. However, the overall influence of age on environmental awareness is not significant (*p* = 0.293). Looking at the mean values of the environmental awareness areas across the occupational groups, no significant differences are apparent. Likewise, the values of the subscales and the overall scale are not significant. The same applies to the influence of gender on environmental awareness (see Table A4).

### 3.3. General Environmental Behavior

Based on the definition that a value of 1 denotes environmentally friendly behavior and a value of 0 denotes environmentally harmful behavior, the average value of the sample (*M* = 0.67, *SD* = 0.10) indicates that the surveyed hospital employees behave in an above-average, environmentally conscious manner. The scores ranged from 0.36 to 0.88. The dispersion around the mean is low (*Var* = 0.01, *SD* = 0.10). No significant outliers were identified (see Table 6).

The overall assessment of the sample as environmentally conscious is supported by the fact that only six respondents (4.6%) had a score below 0.5 and were therefore in the non-environmentally conscious range. With 28 participants (25.4%), more than a quarter of the respondents scored in the upper quartile, whereas no participants were in the lower quartile.

The distributions of the six subscales of the GEB 50 clearly show that the environmental awareness of the respondents varied greatly in the different areas of action: energy saving, mobility, waste prevention, consumption, recycling, and social engagement. While the areas of energy saving, waste prevention, consumption, and recycling achieved higher mean values than the overall scale, the areas of mobility and, in particular, social engagement were below the mean value of the overall scale. The best scores were achieved in the area of recycling behavior. Here, 95 respondents (73.1%) achieved the maximum possible score of 1, indicating that they implemented all of the recycling measures surveyed. A further 29 participants (22.3%) scored in the upper quartile. Only six people were in the lower three quartiles.

The opposite picture emerges from the evaluation of the subscale on social engagement. Not only is the average environmental awareness score approximately 0.38, falling in the range of environmentally harmful behavior, but the majority of surveyed hospital employees also scored below this value, as indicated by the right-skewed distribution. Fifty-two participants (39.3%) scored within the lower quartile of the maximum achievable score. The scores covered the entire spectrum from 0 to 1, indicating that the sample included both participants who responded positively to all questions on social engagement and one individual who answered negatively to all items.

With *r* = 0.67 (*p* < 0.001), a high correlation can be identified between the general environmental behavior measured by the GEB 50 of hospital employees and their general environmental attitudes. This is evident to a slightly lesser extent when taking a closer look at the correlations between the subscales of the GEB 50 and the item batteries of the UBA. Only in the correlation between the overall scale of the GEB 50 and the subscale of intentional environmental behavior can a slightly higher correlation be identified (*r* = 0.75, *p* < 0.001).

### 3.4. Risk Awareness

Risk awareness was measured using two different scales. First, respondents were asked how threatening they considered certain environmental problems to be (item battery F 4.2, UBA 2022). In order to compare the sample from the self-assessment with the representative sample from the UBA survey and to level out the differences described in the surveys, the results were evaluated using the procedure described in Section 2.3.1 and Appendix A. Detailed results are provided in Table A5.

Significant differences between the two surveys are particularly evident in the response option “very threatening.” Hospital employees who participated in the internal survey perceived the threat posed by climate change, species extinction, and the scarcity of raw materials as significantly less “very threatening.” Respondents to the UBA survey, on the other hand, were less likely to view soil pollution, contaminants in food, and ozone depletion as “very threatening.” However, such a clear difference is no longer apparent when looking at the trends, i.e., the summary of the response options into “not (very) threatening” or “very/quite threatening” can no longer be observed. Nevertheless, when comparing the mean values, the differences are significant for at least some questions (*p*_climate change_ = 0.24, *p*_species extinction_ = 0.22, and *p*_raw materials_, *p*_soils_, *p*_food_, *p*_ozone_ < 0.001).

On average, the hospital employees in the internal survey perceived a significantly higher threat potential from the environmental problems mentioned than the respondents in the representative survey (*p* < 0.001), with both surveys showing elevated threat perceptions (*M* = 2.26 and *M* = 2.44, respectively; max = 3). However, no significant differences were observed in the detailed analysis broken down by gender, age, and occupational groups (see Table A4).

With regard to risk awareness, respondents were also asked to assess the direct impact of environmental factors on health. Item battery F 3.1 (UBA, 2022) was used to determine the potential health risks that respondents attributed to certain environmentally harmful factors. The aim was to identify whether there was a difference between the sample of hospital employees and the representative sample of the total population in the Federal Environment Agency’s survey, particularly with regard to these health-related questions. The leveling of the survey differences was also compared here using the procedure described in Section 2.3.1 and in Appendix A.

It was striking that, in the self-assessment, hospital employees in almost all areas were slightly more likely to believe that the environmental factors surveyed could be extremely harmful to health. There were greater differences in the questions about air pollutants and the consequences of climate change. While the mean perceived harm among hospital employees (*M* = 2.14, *SD* = 0.46) was numerically higher than the UBA 2022 reference value (*M* = 2.06), this difference did not reach statistical significance (*t*(129) = 1.89, *p* = 0.060). In contrast, hospital employees attributed a lower risk of harm to the effects of electromagnetic radiation, although no significance could be established (see Table A6). Across both survey groups, the perceived risk of personal health harm was, on average, moderate, with hospital employees providing slightly but non-significantly higher ratings (see Table 7).

The assessment of the risk of damage to health (harm) and environmental attitudes is only slightly correlated at most (*r* = 0.24). The highest values were achieved in terms of environmental cognition (*r* = 0.32), while no correlation could be identified between the assessed risk of harm and intentional environmental behavior (*r* = 0.09). An even weaker connection can be drawn between the environmental behavior of the respondents and their assessment of the risk of damage. Regarding general environmental behavior, only a weak correlation was found (*r* = 0.18).

There is only a moderate correlation between the assessment of the threat potential and general environmental attitudes (*r* = 0.44). Only in the environmental cognition subscale can a low degree of correlation be observed (*r* = 0.20). Similarly, the correlation with general environmental behavior was weak, reaching only *r* = 0.19.

### 3.5. Workplace-Related Environmental Attitudes

A clear majority of the hospital employees expressed support for the seven measures presented in the factorial survey (see Table A7). Disagreement was low, ranging from 0.8% to 11.5% per measure, indicating a high overall level of approval. The measures saving energy (a) and the circular economy (b) received the highest approval ratings.

Attitudes towards the measures surveyed in the hospital were generally positive. For instance, the respondents achieved significantly higher values for workplace-related environmental attitudes with a mean value of 4.41 points compared to the general environmental attitudes with a mean value of 3.67 points (*p* < 0.001). The scores for workplace-related attitudes ranged from 0.95 to 5.00 (*SD* = 0.63; see Table 8).

A strong correlation was found between general and workplace-related environmental attitudes (*r* = 0.62, *p* < 0.01). This relationship remained consistent in the more differentiated analysis according to the three subscales: environmental effect (*r* = 0.60), environmental cognition (*r* = 0.59), and intentional environmental behavior (*r* = 0.42), whereby the relation to environmental behavior is the lowest. All correlations were statistically significant (*p* < 0.01, two-tailed).

### 3.6. Workplace-Related Environmental Behavior

Looking at the willingness to implement the individual workplace-related measures surveyed (see Table 8), it is immediately apparent that the respondents rated their colleagues’ willingness significantly lower than their own. The majority of respondents reported a high to very high willingness to implement the measures surveyed; only a few were unwilling to implement them. The lowest willingness was observed for alternative forms of mobility for commuting (*M* = 3.36), which is consistent with the GEB 50 results for general environmental behavior (see Section 3.2).

In view of the initial thesis of this paper, namely that hospital employees could use their role model function to influence the social transformation process toward greater environmental awareness, question (f) is particularly relevant. Here, the respondents also attested to their colleagues’ comparatively low willingness to convey an environmentally conscious attitude (*M* = 2.51). In contrast, 126 of 130 hospital employees rated their own willingness as high to very high (*M* = 4.36; see Table A8).

On average, respondents achieved a very high score of 4.22 (*SD* = 0.65) in their self-assessment, while it was assumed that colleagues would be significantly less willing to comply with the seven proposed measures (see Table 8). A correlation between one’s own willingness to implement the specified measures in the workplace and the assessment of whether colleagues would be willing to implement them was only found to a small to moderate extent (*r* = 0.38, *p* < 0.01).

There was also only a moderate correlation between workplace-related and general environmental behavior (*r* = 0.43). The correlations with the subscales were even lower, with values between *r* = 0.13 (energy saving) and *r* = 0.34 (mobility). In contrast, workplace-related environmental behavior is highly correlated with general environmental attitudes (*r* = 0.60), which is reflected to a similarly high degree in the sub-areas of environmental affect (*r* = 0.58) and environmental cognition (*r* = 0.55). In the subscale of intentional environmental behavior, analogous to GEB 50, only a moderate correlation can be identified (*r* = 0.42). In contrast, one’s own willingness to implement environmental measures at work correlates very highly with workplace-related environmental attitudes, with *r* = 0.75. All results were statistically significant.

### 3.7. Effectiveness of Measures

The assessment of effectiveness varied depending on the measure presented in the factorial survey. For example, the question about saving unnecessary energy (a) achieved high approval ratings with a mean value of 4.83 and almost no disagreement. In contrast, the effectiveness of hospital employees acting as role models (f) was rated as high by far fewer respondents (*M* = 3.20; see Table A9). The average overall scores for the estimated effectiveness of the measures were close to the average score for the overall survey at *M* = 3.57 (*SD* = 0.55), which aligns with the overall survey mean (see Table 8).

If we correlate the respondents’ assessment of the effectiveness of the measures with their attitudes toward these measures, as reflected in their answers to the question of whether they considered the measures to be appropriate, we can assume a high degree of correlation (*r* = 0.54). The same picture emerges when looking at the correlation with workplace-related environmental behavior, measured in terms of respondents’ willingness to implement the respective measures (*r* = 0.54).

### 3.8. Transformation Costs

In order to convert the average values of the transformation costs into a uniform logic, the values of question 6 were first reversed in their value logic so that agreement with a long implementation period scored lower than disagreement with it. With this approach, the answers to question 6 were harmonized with those of questions 3 and 4 in terms of their statement logic. An overall value for the transformation costs was subsequently calculated. This is characterized by the fact that high values in the transformation costs reflect the assessment that the costs for implementing the measures are low. With a mean value of *M* = 2.56 (*SD* = 0.57), the costs for the transformation were assessed as moderate (see Table A10).

The overall variable of the transformation costs estimated by the respondents was composed of three individual questions. In addition to asking whether the individual measures were easy for employees or the company to implement, respondents were also asked whether this would involve a long implementation period. At first glance, the responses to the individual measures appeared contradictory. For example, the measure “a. In the future, attention should be paid to whether energy is being used unnecessarily” was rated as easy to implement by both the company (*M* = 4.91) and employees (*M* = 4.71). At the same time, however, it was assumed that it would take a long time to implement this measure (mean value of 4.55).

All other measures presented a more homogeneous picture. Measures that were considered less easy to implement were also assigned a slightly longer implementation period. However, only a weak correlation (item 3: *r* = 0.25; item 4: *r* = 0.20) was found between question 6 on the implementation period and questions 3 and 4, which was again highly significant (two-tailed).

The correlation between transformation costs and workplace-related environmental attitudes shows a moderate relationship (*r* = 0.31, *p* < 0.001). The correlation between the assessment of transformation costs and workplace-related environmental behavior, measured in terms of one’s own willingness to implement, was also in the moderate range, albeit slightly higher (*r* = 0.54, *p* < 0.001).

### 3.9. Subjective Social Norms

Table 9 presents the average values across all seven individual items regarding the respondents’ assessments of whether certain environmental standards are specified by hospital management (overall scale). The results indicate that the hospital employees generally expect management to have rather low expectations (*M* = −0.10, *SD* = 0.97). The high dispersion around the mean is reflected in the wide range of individual scores (see Table A11).

The dispersion was high for all questions. On average, respondents tended to assume that questions a, b, and e were expected by company management, albeit to a lesser extent. The four remaining categories of measures were generally not considered to be expected by company management.

The high dispersion is confirmed by a closer look at the frequency distributions (Table A11). The assessments given for the standards expected by company management are distributed fairly evenly across the five response options for all categories of measures. If the two response options “not expected at all” and “probably not expected” as well as “probably expected” and “expected” are combined to form a negative and a positive subjective assessment of the existence of organization-related standards, the picture is the same.

Furthermore, it was investigated whether there is a connection between the norms perceived by employees in the hospital organization—i.e., whether employees assume that certain environmentally related behaviors are expected of them—and other aspects of workplace-related environmental awareness. Specifically, the study examined whether low perceived expectations on the part of management correlate with the following:

a The values of general and workplace-related environmental attitudes are low;
b The willingness to engage in general and workplace-related environmental behavior is low.

No correlation could be observed between the organizational norms and the respondents’ workplace-related environmental attitudes toward the measures listed in the vignettes (*r* = −0.01). The same applies to a correlation between the norms and workplace-related environmental behavior as measured by the respondents’ own willingness to implement them (*r* = −0.01).

### 3.10. Findings via the Structural Equation Model (Path Analysis and Model Fit)

**Figure 2:**
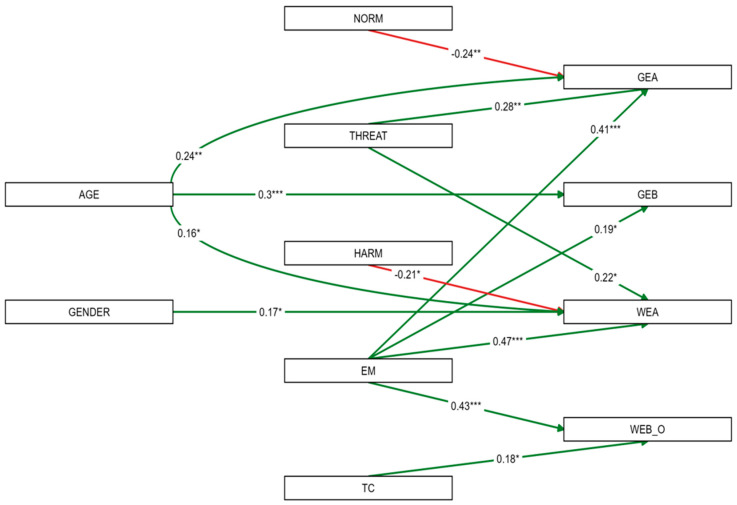
Path model of predictors for environmental attitudes and behavior (N = 130). EM = effectiveness of measure; GEA = general environmental attitudes; GEB = general environmental behavior; NORM = subjective social norms; TC = transformation costs; WEA = workplace-related attitudes; WEB_O = workplace-related behavior. Solid green lines indicate positive significant paths, solid red lines indicate negative significant paths. * *p* < 0.05, ** *p* < 0.01, *** *p* < 0.001.

To test the proposed relationships, a path model was estimated (see Figure 2). As the model was just-identified (saturated), it showed a perfect fit to the data (*Χ*^2^(0) = 0, *p* = 1.000; *CFI* = 1.000; *RMSEA* = 0.000). Standardized coefficients and their significance levels are displayed in Figure 2, where solid green lines indicate positive significant paths and solid red lines indicate negative significant paths. For clarity, non-significant paths are not displayed in the figure.

Effectiveness of measures (EM) emerged as the most consistent and powerful predictor, yielding significant positive paths to all endogenous variables, with the strongest effect on WEA (*β* = 0.468, *p* < 0.001). This could indicate that the belief in the effectiveness of measures is a primary driver of pro-environmental engagement.

Furthermore, the model highlights two notable negative relationships: Social subjective norms are negatively associated with GEA (*β* = 0.236, *p* = 0.001), and perceived harm negatively predicted WEA (*β* = −0.214, *p* = 0.015). In contrast, perceived threat significantly increased both general and workplace-specific attitudes.

The structural model explained significant proportions of variance in all endogenous variables (see Table 10). The highest explained variance was observed for workplace-related environmental attitude (*R*^2^ = 0.392, *p* < 0.001) and general environmental attitude (*R*^2^ = 0.381, *p* < 0.001), while general environmental behavior showed a moderate but significant variance explanation (*R*^2^ = 0.191, *p* = 0.002).

Table 11 shows a summary of standardized path coefficients for the four endogenous variables of the path model and their significant paths (*p* ≤ 0.100).

Regarding demographics, *age* was the strongest unique predictor of actual general environmental behavior (*β* = 0.305, *p* < 0.001). Interestingly, social subjective norms yielded a significant negative path to general attitudes (*β* = −0.236, *p* = 0.001), suggesting potential psychological reactance. Similarly, harm was negatively associated with workplace attitudes (*β* = −0.214, *p* = 0.015), whereas threat showed positive associations with attitudes.

## 4. Discussion

The present study investigated the environmental awareness and behavior of hospital employees in Germany to evaluate their potential role as catalysts for the social transformation toward sustainability. Our results reveal that hospital employees exhibit a high baseline of environmental awareness, significantly surpassing the German general population (Lindenberg, 1985; Beck & Opp, 2001). However, the path analysis (SEM) demonstrates that the transition from awareness to action is moderated by complex psychological and structural predictors, revealing a substantial “knowledge–action gap.”

### 4.1. Predictors of Environmental Attitudes and Behavior

The perceived effectiveness of measures emerged as the most consistent and powerful predictor of environmental attitudes and behavior across all variables, with the strongest effect on workplace-related attitudes (*β* = 0.468, *p* < 0.001), which was also shown by Jenny et al. (2024). It confirms that hospital employees are highly rational actors; their willingness to engage depends primarily on the perceived functional impact of an intervention. This aligns with the “mitigation” focus described in the introduction, where efficacy is a prerequisite for intrinsic motivation.

A critical finding, which is in contrast to other findings (Helferich et al., 2023; Zhang et al., 2026), is the negative association between subjective social norms and general environmental attitudes (*β* = −0.236, *p* = 0.001). This suggests a degree of psychological reactance (Weller, 2024). In the high-pressure environment of German hospitals—currently strained by economic constraints and structural reforms (Hines et al., 1987; Langheine & Lehmann, 1986; Diekmann & Preisendörfer, 1991; Augurzky & Kolodziej, 2018; Augurzky et al., 2025)—top-down social pressure or perceived management expectations may be felt as a threat to individual autonomy, leading to an inner rejection of environmental values. This is further supported by the descriptive result that management expectations were generally perceived as low (*M* = −0.10), and no correlation was found between these norms and actual behavioral willingness (*r* = −0.01). This result, however, is partly in line with other findings from where social norms only played a minor role in implementing green hospitals (Asmawati & Adisasmito, 2024).

### 4.2. Risk Perception and the Barrier of Transformation Costs

The study highlights a nuanced role of risk awareness. While perceived threat positively influenced attitudes, perceived harm negatively predicted workplace attitudes (*β* = −0.214, *p* = 0.015). This suggests that while an immediate sense of threat fosters awareness, focusing on abstract or overwhelming health damage may trigger defensive avoidance mechanisms rather than proactive engagement.

Furthermore, in line with low-cost theory (Ajzen, 1991), transformation costs significantly predicted workplace behavior (*β* = 0.179, *p* = 0.031). Even highly motivated employees are sensitive to the “behavioral costs”—time, effort, and systemic friction—of implementing green measures in a clinical routine. The “better-than-average” effect observed in our data, where respondents rated their own willingness (*M* = 4.22) significantly higher than that of their colleagues (*M* = 2.97), further underscores the perceived social and structural difficulty of achieving a collective behavioral shift.

### 4.3. Implications for Social Tipping Points

Regarding the potential for social tipping points (Dickhoff et al., 2021), our evidence remains inconclusive. Hospital employees possess the necessary awareness and public trust to act as change agents. However, the divergence between intention and behavior, driven by perceived costs and psychological reactance, suggests that the “critical mass” required for a societal rethink has not yet been reached. For this group to trigger a social transformation, institutional support must focus on reducing transformation costs and emphasizing the concrete effectiveness of measures rather than relying on social pressure.

### 4.4. Limitations

Despite the significant findings of this study, several limitations must be acknowledged. First, the sample size (N = 130), while sufficient for the estimation of a path model with the current number of parameters, may limit the statistical power to detect smaller effects, particularly concerning the occupational control groups. Future studies with larger, more diverse samples would be beneficial to enhance the generalizability of the findings across different sectors.

Second, the study utilized a cross-sectional design, meaning that all variables were measured at a single point in time. Consequently, although the path model implies a directional influence from psychological predictors to behavior, no definitive causal inferences can be made. It is possible, for instance, that engaging in general environmental behavior subsequently strengthens one’s environmental attitude through self-perception processes. Longitudinal or experimental designs are needed to confirm the causal pathways suggested by this model.

Third, the data rely on self-reported measures, which may be subject to social desirability bias, particularly regarding ‘green’ behavior and attitudes (Zhu et al., 2024). Participants might have overreported their environmental engagement to align with perceived societal expectations. However, meta-analyses on social desirability in environmental research only found a small effect (Vesely & Klöckner, 2020), and thus, the confounding of the results by social desirability is limited.

Fourth, a potential counterargument to the conclusion that hospital employees indeed have a higher environmental awareness could be that the increased values are due to the time lag between the survey waves (2018/2022 and 2024). However, this can be refuted by the fact that the development over time does not necessarily lead to a general increase in environmental awareness. Looking at the development of the values in the representative survey, it is noticeable that these values declined in all three areas (environmental impact, environmental cognition, and environmental behavior) between 2018 and 2022. This trend is further supported by the fact that the proportion of respondents who stated in the representative surveys that they consider environmental and climate protection to be an important issue has fallen from 65% to 54% in recent years up to 2024 (Frick et al., 2025).

Finally, the unexpected negative relationship between social subjective norms and attitudes suggests a potential reactance effect specific to this sample or organizational culture. Future research should investigate whether high levels of perceived external pressure in professional environments might inadvertently trigger defensive responses, thereby hindering the internalization of environmental values.

A limitation of the present study is the internal consistency of the transformation costs scale (*α* = 0.67). Although this value is often considered sufficient, it reflects the complexity of economic and structural barriers in the healthcare sector. Future research should therefore aim to refine this instrument to enhance measurement reliability.

## 5. Conclusions

This study demonstrates that hospital employees in Germany exhibit high levels of environmental awareness and a strong belief in the effectiveness of mitigation measures. However, the path to consistent pro-environmental behavior is obstructed by structural and psychological barriers. The path analysis reveals that while perceived threat and effectiveness foster positive attitudes, subjective social norms can provoke reactance, and transformation costs act as a persistent bottleneck for implementation.

The hypothesis that healthcare workers can currently serve as primary drivers of social transformation is only partially supported. While they possess the reputational capital and awareness, the structural constraints of the healthcare system prevent this potential from manifesting as transformative action. To activate this group as key players, we propose the following targeted policy and management interventions:

1. Reduction in Individual Transformation Costs: Policymakers must move beyond mere appeals to intrinsic motivation. We recommend the implementation of a “green default” infrastructure within hospitals (e.g., automated waste separation systems, energy-saving clinical pathways) that reduces the cognitive and temporal burden on staff.
2. Incentive Systems and Regulatory Frameworks: Aligning with the IPCC’s assessment that individual motivation alone is insufficient (Watson, 2001), a national regulatory framework is needed that integrates sustainability into hospital financing and quality management. This should include financial incentives for carbon-neutral procurement and the reduction in medical waste.
3. Reframing Social Norms to Avoid Reactance: Management strategies should pivot from top-down moral appeals—which our data shows can trigger resistance—toward participatory “bottom-up” initiatives. Establishing “Green Teams” or sustainability ambassadors within clinical departments can transform social norms from perceived pressure into a shared professional identity.
4. Strengthening Reputational Leverage: Health authorities should utilize the high public trust in healthcare professionals by providing them with evidence-based toolkits for patient communication regarding climate-health links. This empowers staff to act as multipliers for social tipping points without overextending their clinical resources.

In conclusion, only by aligning ecological requirements with the clinical and economic reality of the hospital environment can healthcare professionals help reach the social tipping points necessary for a sustainable future. By shifting the focus from individual responsibility to systemic enabling, the healthcare sector can transition from a resource-intensive industry to a frontrunner of social-ecological transformation.

This aligns with the assessment of the IPCC, which noted as early as 2001 that, at a minimum, an incentive system—and ideally a regulatory framework—is needed to support mitigation efforts, including changes in individual behavior. Personal motivation based on attitudes alone is therefore insufficient. Based on this insight, there is a need for the political will to provide such a regulatory framework that addresses not only technological adaptation but also mitigation in individual behavior. Hospital staff and healthcare professionals, in general, can then play a role in fostering acceptance of such regulations among the public.

## Figures and Tables

**Table 1:** Matrix for the exemplary classification of environmental measures.

Area of Action	Category of Measures	Example of Measures
Influence of health professions on changes in awareness among the population	Direct communication	“Climate consultation”, political engagement
	Indirect communication	Environmentally conscious behavior as a role model
Structural and infrastructural adjustments	Building refurbishment	Insulation, renewable energies, building materials
	Internal infrastructure	Ventilation and cooling systems, digitalization
Process improvements	Changes in cross-sectional areas	Energy saving, circular economy, catering, mobility
Optimization in care and therapy	Reduction in greenhouse gas emissions	Anesthetic gases
	Saving resources and materials	Medication savings, material savings
	Avoiding non-indicated healthcare services	Avoiding over- and under-treatment, increasing efficiency
Supply chains	Environmentally conscious product selection	Consideration of standards for direct suppliers, consideration of standards along the entire supply chain

**Table 2:** Factorial survey—measures for environmental and climate protection in hospitals.

Please Imagine That Your Company Management Has Recently Been Increasingly Concerned With So-Called Green Hospital Measures in Terms of Sustainability. For This Purpose, a Process Is Being Initiated to Find Out Which Measures Can Be Classified as Target-Oriented and Realistically Implementable. Therefore, the Employees’ Opinions Are Also an Important Influencing Factor.
a. In the future, attention should be paid to whether energy is being used unnecessarily.
b. Measures are to be introduced to exploit potential along the circular economy.
c. It is decided to switch the canteen food and patient catering to a more plant-based diet.
d. Employees should use bicycles or public transportation to commute to work whenever possible.
e. Stockpiling of medicines should be reduced, and instead, they should be ordered more frequently and in smaller packaging sizes.
f. Employees should convey an environmentally conscious attitude (role model function).
g. In the future, greater attention will be paid to the CO_2_ footprint and environmentally friendly production of the materials used along the supply chain.

**Table 3:** Factorial survey—assessment of the measures for environmental and climate protection in hospitals.

Measures	I Completely Disagree	I Disagree	I Agree	I Completely Agree
1. I think this decision is right.				
2. I think the measures are having a significant effect.				
3. I think the measures are easy for the company to implement.				
4. I think the measures are easy for employees to implement.				
5. I think most colleagues would adhere to these guidelines.				
6. I think it will take quite a long time to implement/enforce these measures across the board.				
7. I am happy to implement the measure or am already doing so.				

*Note.* Workplace-related environmental attitudes were measured using question 1; workplace-related behavior was measured using questions 5 and 7; effectiveness was measured using question 2; the transformation costs were measured using questions 3, 4, and 6.

**Table 4:** Total sample subdivided by gender, age, and occupational group.

Characteristic	Total *n* (%)	Female *n* (%)	Male *n* (%)
Total Sample	130 (100.00%)	94 (72.31%)	36 (27.69%)
Age			
≤30	15 (11.54%)	11 (8.46%)	4 (3.08%)
31–40	43 (33.08%)	34 (26.15%)	9 (6.92%)
41–50	38 (29.23%)	25 (19.23%)	13 (10.00%)
51–60	25 (19.23%)	18 (13.85%)	7 (5.38%)
>60	9 (6.92%)	6 (4.62%)	3 (2.31%)
Occupational Group			
Medical services	55 (42.31%)	35 (15.38%)	20 (26.92%)
Administrative staff	23 (17.69%)	15 (11.54%)	8 (6.15%)
Nursing staff	21 (16.15%)	17 (13.08%)	4 (3.08%)
Others	31 (23.85%)	27 (20.77%)	4 (3.08%)

**Table 5:** General environmental attitudes—descriptive results and comparison with UBA data.

Variable	UBA 2018	UBA 2022	GHS 2025	Min	Max	*p*-Value	*p*-Value
	Mean	Mean	Mean (*SD*)			(vs. 2018)	(vs. 2022)
GEA	3.24	3.16	3.67 (0.68)	1.39	4.91	<0.001	<0.001
EE	3.58	3.43	4.17 (0.81)	1.19	5	<0.001	<0.001
EC	3.93	3.75	4.03 (0.67)	1.67	5	0.088	<0.001
EB	2.31	2.3	2.87 (0.91)	0.38	4.75	<0.001	<0.001

*Note.* General environmental attitudes (aggregated); EE = environmental effect; EC = environmental cognition; EB = environmental behavior (intentional). GHS 2025 = Green Hospital Survey (present study).

**Table 6:** General ecological behavior scale—descriptive results.

Variable	Median	Mean (*SD*)	Min	Max	Range
GEB 50	0.68	0.67 (0.10)	0.36	0.88	0.52
ES	0.82	0.77 (0.13)	0.45	1	0.55
M	0.58	0.56 (0.15)	0.25	0.92	0.67
WP	1	0.89 (0.14)	0.4	1	0.6
C	0.7	0.71 (0.17)	0.2	1	0.8
RC	1	0.92 (0.15)	0.25	1	0.75
SE	0.38	0.40 (0.20)	0	1	1

*Note.* GEB 50 = General ecological behavior scale; ES = energy saving, M = mobility cognition, WP = waste prevention, C = consumption, RC = recycling, SE = social engagement.

**Table 7:** Risk awareness—descriptive results.

	Mean (*SD*)	Min	Max	Range
RA (threat)—GHS	2.44 (0.41)	0.87	3.00	2.13
UBA 2022 (threat)	2.26			
RA (harm)—GHS	2.14 (0.46)	0.89	3.00	2.11
UBA 2022 (harm)	2.06	0.40	1.00	0.60

*Note.* RA = risk awareness; GHS = green hospital survey (present study). UBA 2022 data rescaled for comparability (see Appendix A).

**Table 8:** Descriptive results of the factorial survey and aggregated scales.

	Median	Mean (*SD*)	Min	Max	Range
S	3.59	3.55 (0.46)	1.98	4.65	2.67
WEA	4.52	4.41 (0.63)	0.95	5.00	4.05
WEB_C	2.98	2.97 (0.65)	1.39	5.00	3.61
WEB_O	4.29	4.22 (0.65)	1.91	5.00	3.09
EM	3.57	3.57 (0.55)	2.08	4.76	2.68
TC	2.62	2.56 (0.57)	1.03	4.21	3.17

*Note.* S = Aggregated survey results; WEA = workplace-related attitudes; WEB_C = workplace-related environmental behavior (willingness of colleagues); WEB_O = workplace-related environmental behavior (own willingness); EM = effectiveness of measures; TC = transformation costs.

**Table 9:** Subjective norms—descriptive results of perceived management expectations.

(5) Our Management Expects the Responsible Employees to	Median	Mean (*SD*)	Min	Max	Range
Subjective Norms (aggregated)	0	−0.10 (0.97)	−2	2	4
a. actively try to save energy (e.g., by opening windows for ventilation, turning off lights in unused areas).	1	0.50 (1.38)	−2	2	4
b. exploit as much potential as possible for energy and resource savings in the circular economy (e.g., reduction in consumables).	1	0.24 (1.36)	−2	2	4
c. focus on sustainable catering for employees and patients (e.g., plant-based nutrition, seasonal products, optimized portion sizes).	−1	−0.72 (1.25)	−2	2	4
d. try to reduce emissions by changing mobility habits (e.g., commuting by bicycle or public transport).	−1	−0.33 (1.35)	−2	2	4
e. avoid unnecessary medication waste (e.g., through optimized ordering processes or smaller packaging units).	0	0.35 (1.24)	−2	2	4
f. set an example through environmentally conscious behavior.	0	−0.23 (1.43)	−2	2	4
g. select products that take sustainability standards in the supply chain into account.	0	−0.50 (1.17)	−2	2	4

**Table 10:** Summary of explained variance (*R*^2^) for endogenous variables (N = 130).

Variable	*R* ^2^	SE	z	*p*-Value
GEA	0.381	0.067	5.678	<0.001
GEB	0.191	0.062	3.084	0.002
WEA	0.392	0.067	5.875	<0.001
WEB_O	0.367	0.067	5.459	<0.001

**Table 11:** Standardized path coefficients of environmental attitudes and behaviors (sorted by significance).

Outcome	Predictor	β	SE	z	*p*-Value
GEB	Age	0.305	0.081	3.764	<0.001
	EM	0.186	0.094	1.985	0.047
	NORM	−0.144	0.085	−1.692	0.091
GEA	EM	0.406	0.079	5.124	<0.001
	THREAT	0.283	0.087	3.251	0.001
	NORM	−0.236	0.074	−3.189	0.001
	Age	0.240	0.073	3.297	0.001
WEA	EM	0.468	0.077	6.099	<0.001
	THREAT	0.223	0.087	2.564	0.010
	HARM	−0.214	0.088	−2.428	0.015
	Gender (1 = female)	0.171	0.073	2.343	0.019
	Age	0.164	0.073	2.256	0.024
WEB_O	EM	0.428	0.079	5.428	<0.001
	TC	0.179	0.083	2.159	0.031
	THREAT	0.143	0.089	1.609	0.108

*Note.* Non-significant paths (*p* > 0.10) are omitted for brevity.

## Data Availability

The original contributions presented in this study are included in the article. Further inquiries can be directed to the corresponding author.

## Abbreviations

The following abbreviations are used in this manuscript:

C	Consumption
CFI	Comparative fit index
CO_2_	Carbon dioxide
EB	Environmental behavior (intentional)
EC	Environmental cognition
EE	Environmental effect
EM	Effectiveness of measures
ES	Energy saving
GEA	General environmental attitude
GEB	General environmental behavior
GEB 50	General ecological behavior scale
GHS	Green hospital survey
IPCC	Intergovernmental panel on climate change
KHVVG	Krankenhausversorgungsverbesserungsgesetz (Hospital Services Improvement Act)
M	Mobility cognition
ML	Maximum likelihood
RA	Risk awareness
RC	Recycling
RMSEA	Root mean square error of approximation
SE	Social engagement
SEM	Structural equation modeling
TC	Transformation costs
UBA	German Environment Agency (Umweltbundesamt)
UNFCCC	United Nations framework convention on climate change
WEA	Workplace-related environmental attitude
WEB-C	Workplace-related environmental behavior (willingness of colleagues)
WEB-O	Workplace-related environmental behavior (own willingness)
WP	Waste prevention

## Appendix A

F 3.1 (harm): In the self-assessment survey, respondents were mistakenly not given the option of responding with a middle, “neutral” scale value (“moderate harm”), meaning that they always had to choose one side of the spectrum. A summary evaluation was therefore carried out for both surveys. For the evaluation of the frequency distribution, the responses with a recognizable tendency were summarized (“(extreme) harm” or “some/no harm”). The results of the original survey were transformed so that the neutral responses given (“moderate harm”) were divided evenly between the two tendencies. In order to make the mean values of the two surveys comparable, the response options of the different scales were assigned different scale values, with a value of 0 being assigned to the response option “no harm” and a value of 3 being assigned to the response option “extreme harm” (see Table A1).

**Table A1:** Risk awareness (harm)–coding scheme.

Variable	Value Label Coded (Own Survey)	Value Label Coded (UBA 2022)
Pollutants and pesticide residues in food	0 = no harm 1 = some harm 2 = significant harm 3 = extreme harm	0 = no harm 0.75 = some harm 1.5 = moderate harm 2.25 = significant harm 3 = extreme harm
Chemicals in everyday products and items		
Electromagnetic radiation from cell phones, tablets, and computers		
Electromagnetic radiation from cell phone towers		
Pollutants in drinking water		
Plastic particles in drinking water and food		
Air pollutants		
Noise		
Consequences of climate change (e.g., heat waves, floods)		

F 4.2 (threat): In the survey on perceived threat levels, the problem of reversed response options was exactly the opposite. This time, the self-assessment survey included one more option than the comparative study by the Federal Environment Agency, namely the neutral response option “moderately threatening.” While the UBA’s representative survey required respondents to choose a particular tendency in their statements, the respondents in the self-assessment survey this time had the option of giving a neutral response. Therefore, for the evaluation of the frequency distribution, the responses with a recognizable tendency were again summarized, whereby in this case the results of the self-assessment were transformed so that the neutral responses given were distributed evenly between both tendencies (see Table A2).

**Table A2:** Risk awareness (threat)–coding scheme.

Variable	Value Label Coded (Own Survey)	Value Label Coded (UBA 2022)
Climate change	0 = not threatening 0.75 = not very threatening 1.5 = moderately threatening 2.25 = quite threatening 3 = very threatening	0 = not threatening 1 = not very threatening 2 = somewhat threatening 3 = very threatening
Species extinction in the animal and plant world		
Condition of forests (due to deforestation, drought, storm damage, etc.)		
Pollutant levels in soil, water, and air		
Plastic waste and plastic pollution in nature (e.g., oceans, soil)		
Contaminants in food		
Shortage of raw materials (such as oil, metals, sand, rare earths, etc.)		
Land consumption through soil sealing (e.g., through building and road construction)		
Shortage of fresh water reserves		
Ozone depletion, ozone holes		
Ocean acidification		
Environmental impact of intensive livestock farming		
Environmental pollution caused by overfertilization (e.g., through the excessive use of manure and mineral fertilizers in agriculture)		

**Table A3:** Subjective norms–coding scheme.

Our Management Expects the Responsible Employees to	Value Label Coded (Own Survey)
1. actively try to save energy (e.g., by opening windows for ventilation, turning off lights in unused areas).	−2 = not expected at all −1 = not really expected 0 = I don’t know if it’s expected 1 = somewhat expected 2 = definitely expected
2. exploit as much potential as possible for energy and resource savings in the circular economy (e.g., reduction of consumables).	
3. focus on sustainable catering for employees and patients (e.g., plant-based nutrition, seasonal products, optimized portion sizes).	
4. try to reduce emissions by changing mobility habits (e.g., commuting by bicycle or public transport).	
5. avoid unnecessary medication waste (e.g., through optimized ordering processes or smaller packaging units).	
6. set an example through environmentally conscious behavior.	
7. select products that take sustainability standards in the supply chain into account.	

**Table A4:** General environmental attitudes–comparison of mean values by gender, age, and profession.

Variable	GEA Mean (± *SD*)	EE Mean (± *SD*)	EC Mean (± *SD*)	EB Mean (± *SD*)
Gender				
Female	3.70 (± 0.62)	4.22 (± 0.76)	4.10 (± 0.66)	2.84 (± 0.82)
Male	3.60 (± 0.84)	4.03 (± 0.95)	3.86 (± 0.74)	2.95 (± 1.10)
*p-value*	*0.438*	*0.234*	*0.073*	*0.538*
Age				
≤30	3.56 (± 0.86)	4.16 (± 1.03)	3.96 (± 0.82)	2.64 (± 1.03)
31–40	3.65 (± 0.57)	4.21 (± 0.68)	4.01 (± 0.64)	2.81 (± 0.84)
41–50	3.60 (± 0.76)	4.04 (± 1.00)	3.98 (± 0.74)	2.84 (± 0.94)
51–60	3.70 (± 0.65)	4.19 (± 0.67)	4.11 (± 0.69)	2.85 (± 0.89)
>60	4.13 (± 0.50)	4.52 (± 0.46)	4.26 (± 0.45)	3.67 (± 0.69)
*p-value*	*0.293*	*0.605*	*0.803*	*0.085*
Occupation				
Medical services	3.65 (± 0.67)	4.18 (± 0.86)	3.93 (± 0.66)	2.89 (± 0.88)
Administrative staff	3.75 (± 0.71)	4.21 (± 0.63)	4.14 (± 0.65)	2.97 (± 1.09)
Nursing staff	3.65 (± 0.54)	4.11 (± 0.73)	4.07 (± 0.52)	2.84 (± 0.73)
Others	3.67 (± 0.79)	4.17 (± 0.93)	4.11 (± 0.85)	2.77 (± 0.94)
*p-value*	*0.940*	*0.984*	*0.556*	*0.884*

*Note.* GEA = general environmental attitudes (aggregated); EE = environmental effect, EC = environmental cognition; EB = environmental behavior.

**Table A5:** Frequency distributions for risk awareness (threat).

Variable	Very Threatening	Quite Threatening	Moderately Threatening	Not Very Threatening	Not Threatening	Very/Quite Threatening	Not (Very) Threatening
Climate change	56	53	15	3	3	109	6
	43.10%	40.80%	11.50%	2.30%	2.30%	89.65%	10.35%
UBA 2022	59.18%	30.61%		8.15%	2.04%	89.79%	10.20%
Species extinction in the animal and plant world	52	49	22	6	1	101	7
	40.00%	37.70%	16.90%	4.60%	3.80%	86.15%	13.85%
UBA 2022	52.04%	41.84%		9.18%	1.02%	89.80%	10.20%
Condition of forests (due to deforestation, drought, storm damage, etc.)	60	58	6	5	1	118	6
	46.20%	44.60%	4.60%	3.80%	0.80%	93.10%	6.90%
UBA 2022	52.04%	41.84%		5.10%	1.02%	93.88%	6.12%
Pollutant levels in soil, water, and air	84	40	5	1	0	124	1
	64.60%	30.80%	3.80%	0.80%	0.00%	97.30%	2.70%
UBA 2022	42.86%	47.96%		7.14%	1.02%	90.82%	8.16%
Plastic waste and plastic pollution in nature (e.g., oceans, soil)	67	42	5	1	0	124	1
	51.50%	32.30%	13.80%	2.30%	0.00%	90.70%	9.20%
UBA 2022	62.24%	32.65%		4.08%	1.02%	94.82%	8.16
Contaminants in food	86	31	10	2	1	117	3
	66.20%	23.80%	7.70%	1.50%	0.80%	93.85%	6.15%
UBA 2022	41.84%	46.94%		10.20%	1.02%	88.78%	11.22%
Shortage of raw materials (such as oil, metals, sand, rare earths, etc.)	24	38	43	17	8	62	25
	18.50%	29.20%	33.10%	13.10%	6.20%	64.25%	35.85%
UBA 2022	29.59%	50.00%		17.35%	1.02%	79.59%	18.37%
Land consumption through soil sealing (e.g., through building and road construction)	44	48	28	8	2	92	10
	33.80%	36.90%	21.50%	6.20%	1.50%	81.45%	18.45%
UBA 2022	27.55%	52.04%		16.33%	1.02%	79.59%	17.35%
Shortage of fresh water reserves	89	33	7	1	0	122	10
	68.50%	25.40%	5.40%	0.80%	0.00%	96.60%	10.70%
UBA 2022	60.20%	32.65%		5.10%	1.02%	92.85%	6.12%
Ozone depletion, ozone holes	70	39	17	3	1	109	4
	53.80%	30.00%	13.10%	2.30%	0.80%	90.35%	9.65%
UBA 2022	30.61%	49.90%		14.29%	2.04%	77.51%	16.33%
Ocean acidification	69	45	43	2	1	114	3
	53.10%	34.60%	10.00%	1.50%	0.80%	92.70%	7.30%
UBA 2022	39.80%	44.90%		7.14%	1.02%	84.70%	8.16%
Environmental impact of intensive livestock farming	52	55	19	3	1	107	4
	40.00%	42.30%	14.60%	2.30%	0.80%	89.60%	10.40%
UBA 2022	26.60%	48.98%		20.41%	2.04%	75.58%	22.45%
Environmental pollution caused by overfertilization (e.g., through the excessive use of manure and mineral fertilizers in agriculture)	70	46	10	4	0	116	4
	53.80%	35.40%	7.70%	3.10%	0.00%	93.05%	6.95%
UBA 2022	32.65%	50.00%		12.24%	2.04%	82.65%	14.28%

**Table A6:** Frequency distributions for risk awareness (harm).

Variable	Extreme Harm	Significant Harm	Moderate Harm	Some Harm	No Harm	(Extreme) Harm	Some/No Harm
Pollutants and plastic residues in food	60	58		12	10	118	12
	46.20%	44.6%		9.20%	0.00%	90.8%	9.20%
UBA 2022	42.86%	37.76%	13.27%	5.10%	1.02%	87.26%	12.76%
Chemicals in everyday products and items	60	51		17	2	111	19
	46.20%	39.20%		13.10%	1.50%	85.40%	14.60%
UBA 2022	37.76%	38.78%	16.33%	6.12%	1.02%	84.71%	15.31%
Electromagnetic radiation from cell phones, tablets, and computers	8	38		62	22	46	84
	6.20%	29.20%		47.70%	16.90%	35.40%	64.60%
UBA 2022	6.25%	18.75%	33.33%	23.96%	17.71%	41.67%	58.34%
Electromagnetic radiation from cell towers	7	34		64	25	41	89
	5.40%	26.20%		49.20%	19.20%	31.60%	68.40%
UBA 2022	7.45%	17.02%	33.98%	22.34%	20.21%	40.96%	59.04%
Pollutants in drinking water	78	38		13	1	116	14
	60.00%	29.20%		10.00%	0.80%	89.20%	10.80%
UBA 2022	48.98%	33.67%	11.22%	4.08%	2.04%	88.26%	11.73%
Plastic particles in drinking water and food	67	51.5		9.2	1	118.5	10.2
	51.50%	38.5%		9.20%	0.80%	90.00%	10.00%
UBA 2022	44.90%	35.71%	13.27%	5.10%	1.02%	87.25%	12.76%
Air pollutants	74	52		74	0	126	4
	56.90%	40.00%		3.10%	0.00%	96.90%	3.20%
UBA 2022	32.65%	42.86%	18.37%	5.10%	1.02%	84.70%	15.31%
Noise	32	71		26	1	103	27
	24.60%	54.60%		20.00%	0.80%	79.20%	20.80%
UBA 2022	19.19%	40.40%	27.27%	11.11%	2.02%	73.23%	26.77%
Consequences of climate change	93	32		4	1	125	5
	71.50%	24.60%		3.10%	0.80%	96.10%	3.90%
UBA 2022	35.71%	37.76%	27.35%	7.14%	2.04%	82.15%	17.86%

**Table A7:** Frequency distributions for workplace-related environmental attitudes.

Variable	Completely Disagree	Tend to Disagree	Tend to Agree	Completely Agree	Mean (*SD*)
a. In future, attention should be paid to whether energy is being used unnecessarily.	0	2	18	110	4.72
	0.0%	1.5%	13.8%	84.6%	(0.69)
b. Measures should be introduced to exploit potential along the circular economy.	1	0	19	110	4.72
	0.8%	0.0%	14.6%	84.6%	(0.72)
c. It is decided to switch the canteen food and patient meals to a more plant-based diet.	3	12	22	93	4.29
	2.3%	9.2%	16.9%	71.5%	(1.26)
d. Employees should commute to work by bicycle or public transportation whenever possible.	2	7	30	91	4.36
	1.5%	5.4%	23.1%	70.0%	(1.11)
e. The stockpiling of medicines should be reduced and instead orders should be placed more frequently and in smaller package sizes.	1	12	49	57	3.94
	0.8%	9.2%	37.7%	51.5%	(1.16)
f. Employees should convey an environmentally conscious attitude (role model function).	0	4	34	92	4.46
	0.0%	3.1%	26.2%	70.8%	(0.89)
g. In future, greater attention will be paid to the carbon footprint and environmentally friendly production of the materials used along supply chains.	1	5	43	81	4.28
	0.8%	3.8%	33.1%	62.3%	(1.02)

**Table A8:** Frequency distributions for workplace-related environmental behavior.

**I think most colleagues would adhere to these guidelines.**	**Completely disagree**	**Tend to disagree**	**Tend to agree**	**Completely agree**	**Mean** **(*SD*)**
a. In future, attention should be paid to whether energy is being used unnecessarily.	0	4	43	83	4.35 (0.92)
	0.0%	3.1%	33.1%	63.8%	
b. Measures should be introduced to exploit potential along the circular economy.	3	33	80	14	3.01 (1.08)
	2.3%	25.4%	61.5%	10.8%	
c. It is decided to switch the canteen food and patient meals to a more plant-based diet.	7	62	50	11	2.50 (1.21)
	5.4%	47.7%	38.5%	8.5%	
d. Employees should commute to work by bicycle or public transportation whenever possible.	17	73	32	8	2.06 (1.26)
	13.1%	56.2%	24.6%	6.2%	
e. The stockpiling of medicines should be reduced and instead orders should be placed more frequently and in smaller package sizes.	2 1.5%	25 19.2%	68 52.3%	22 25.4%	3.39 (1.20)
f. Employees should convey an environmentally conscious attitude (role model function).	5	63	52	9	2.51 (1.14)
	3.8%	48.5%	40.0%	6.9%	
g. In future, greater attention will be paid to the carbon footprint and environmentally friendly production of the materials used along supply chains.	5 3.8%	27 20.8%	85 65.4%	13 10.0%	3.03 (1.09)
**I am happy to implement the measure or am already doing so.**	**Completely disagree**	**Tend to disagree**	**Tend to agree**	**Completely agree**	**Mean** **(*SD*)**
a. In future, attention should be paid to whether energy is being used unnecessarily.	0	0	1	129	4.91 (0.37)
	0.0%	0.00%	0.8%	99.2%	
b. Measures should be introduced to exploit potential along the circular economy.	1	1	22	106	4.65 (0.80)
	0.8%	0.8%	16.9%	81.5%	
c. It is decided to switch the canteen food and patient meals to a more plant-based diet.	7	14	27	82	4.03 (1.48)
	5.4%	10:85	20:85	63:15	
d. Employees should commute to work by bicycle or public transportation whenever possible.	13	26	37	54	3.36 (1.69)
	10.0%	20.0%	28.5%	41.5%	
e. The stockpiling of medicines should be reduced and instead orders should be placed more frequently and in smaller package sizes.	1 0.8%	8 6.2%	46 35.4%	73 56.2%	4.15 (1.09)
f. Employees should convey an environmentally conscious attitude (role model function).	0	4	42	84	4.36 (0.91)
	0.00%	3.1%	32.3%	64.6%	
g. In future, greater attention will be paid to the carbon footprint and environmentally friendly production of the materials used along supply chains.	2 1.5%	9 6.9%	52 40.0%	67 51.5%	4.03 (1.15)

**Table A9:** Frequency distributions for effectiveness of measures.

I Think the Measures Are Having a Significant Effect	Completely Disagree	Tend to Disagree	Tend to Agree	Completely Agree	Mean (*SD*)
a. In future, attention should be paid to whether energy is being used unnecessarily.	0	1	11	118	4.83 (0.54)
	0.0%	0.8%	8.5%	90.8%	
b. Measures should be introduced to exploit potential along the circular economy.	1	6	45	77	4.22 (1.04)
	0.8%	4.6%	34.6%	59.2%	
c. It is decided to switch the canteen food and patient meals to a more plant-based diet.	2	11	49	68	4.01 (1.19)
	1.5%	8.5%	37.7%	52.3%	
d. Employees should commute to work by bicycle or public transportation whenever possible.	2	10	40	78	4.15 (1.18)
	1.5%	7.7%	30.8%	60.0%	
e. The stockpiling of medicines should be reduced and instead orders should be placed more frequently and in smaller package sizes.	3 2.3%	25 19.2%	53 40.8%	48 36.9%	3.55 (1.34)
f. Employees should convey an environmentally conscious attitude (role model function).	3	41	48	37	3.20 (1.39)
	2.3%	31.5%	69.9%	28.5%	
g. In future, greater attention will be paid to the carbon footprint and environmentally friendly production of the materials used along supply chains.	3 2.3%	10 7.7%	52 40.0%	64 49.2%	3.95 (1.22)

**Table A10:** Frequency distributions for transformation costs.

**I think the measures are easy for the company to implement.**	**Completely disagree**	**Tend to disagree**	**Tend to agree**	**Completely agree**	**Mean** **(*SD*)**
a. In future, attention should be paid to whether energy is being used unnecessarily.	0	1	20	109	4.72 (0.66)
	0.0%	0.8%	15.4%	83.8%	
b. Measures should be introduced to exploit potential along the circular economy.	2	45	66	17	2.92 (1.16)
	1.5%	34.6%	50.8%	13.1%	
c. It is decided to switch the canteen food and patient meals to a more plant-based diet.	7	49	46	28	2.88 (1.43)
	5.4%	37.7%	35.4%	21.5%	
d. Employees should commute to work by bicycle or public transportation whenever possible.	7	35	52	36	3.17 (1.45)
	5.4%	26.9%	40.0%	27.7%	
e. The stockpiling of medicines should be reduced and instead orders should be placed more frequently and in smaller package sizes.	6 4.6%	46 35.4%	49 37.7%	28 24.5%	2.95 (1.41)
f. Employees should convey an environmentally conscious attitude (role model function).	7	49	49	25	2.85 (1.40)
	5.4%	37.7%	37.7%	19.2%	
g. In future, greater attention will be paid to the carbon footprint and environmentally friendly production of the materials used along supply chains.	20 15.4%	61 46.9%	34 26.2%	15 11.5%	2.23 (1.46)
**I think the measures are easy for employees to implement.**	**Completely disagree**	**Tend to disagree**	**Tend to agree**	**Completely agree**	**Mean** **(*SD*)**
a. In future, attention should be paid to whether energy is being used unnecessarily.	0	1	5	124	4.91 (0.43)
	0.0%	0.8%	3.8%	95.4%	
b. Measures should be introduced to exploit potential along the circular economy.	3	33	67	27	3.18 (1.24)
	2.3%	25.4%	51.5%	20.8%	
c. It is decided to switch the canteen food and patient meals to a more plant-based diet.	9	29	57	35	3.18 (1.46)
	6.9%	22.3%	43.8%	26.9%	
d. Employees should commute to work by bicycle or public transportation whenever possible.	14	56	50	10	2.38 (1.31)
	10.8%	43.1	38.5%	7.7%	
e. The stockpiling of medicines should be reduced and instead orders should be placed more frequently and in smaller package sizes.	4 3.1%	34 26.2%	61 46.9%	30 32.1	3.18 (1.31)
f. Employees should convey an environmentally conscious attitude (role model function).	3	43	67	17	2.92 (1.18)
	2.3%	33.1%	51.5%	13.1%	
g. In future, greater attention will be paid to the carbon footprint and environmentally friendly production of the materials used along supply chains.	16 12.3%	53 40.8%	49 37.7%	12 9.2%	2.40 (1.38)
**I think it will take quite a long time to enforce these measures across the board.**	**Completely disagree**	**Tend to disagree**	**Tend to agree**	**Completely agree**	**Mean** **(*SD*)**
a. In future, attention should be paid to whether energy is being used unnecessarily.	0	3	29	98	4.55 (0.82)
	0.0%	2.3%	22.3%	75.4%	
b. Measures should be introduced to exploit potential along the circular economy.	1	26	61	42	3.51 (1.23)
	0.8%	20.0%	46.9%	32.3%	
c. It is decided to switch the canteen food and patient meals to a more plant-based diet.	7	29	56	38	3.27 (1.43)
	5.4%	22.3%	43.1%	29.2%	
d. Employees should commute to work by bicycle or public transportation whenever possible.	7	19	57	47	3.51 (1.41)
	5.4%	14.6%	43.8%	36.2%	
e. The stockpiling of medicines should be reduced and instead orders should be placed more frequently and in smaller package sizes.	6 4.6%	34 26.2%	54 41.5%	34 26.2%	3.18 (1.41)
f. Employees should convey an environmentally conscious attitude (role model function).	1	31	61	37	3.38 (1.24)
	80.0%	23.8%	46.9%	28.5%	
g. In future, greater attention will be paid to the carbon footprint and environmentally friendly production of the materials used along supply chains.	3 2.3%	17 13.1%	64 49.2%	46 34.4%	3.63 (1.24)

**Table A11:** Frequency distributions for subjective social norms.

Our Management Expects the Responsible Employees to	Not Expected at All	Probably Not Expected	Don’t Know If It’s Expected	Probably Expected	Expected	Not Expected	Expected	Mean (*SD*)
a. actively try to save energy (e.g., by opening windows for ventilation, turning off lights in unused areas).	13 10.0%	27 20.8%	12 9.2%	38 29.2%	40 30.8%	40 30.8%	78 60.0%	0.50 (1.38)
b. exploit as much potential as possible for energy and resource savings in the circular economy (e.g., reduction of consumables).	18 13.8%	29 22.3%	11 8.5%	48 39.9%	24 18.5%	40 30.8%	72 58.4%	0.24 (1.36)
c. focus on sustainable catering for employees and patients (e.g., plant-based nutrition, seasonal products, optimized portion sizes).	45 34.6%	39 30.0%	17 13.1%	22 16.9%	7 5.4%	84 64.6%	29 22.3%	−0.72 (1.25)
d. try to reduce emissions by changing mobility habits (e.g., commuting by bicycle or public transport).	31 23.8%	42 32.3%	7 5.4%	29 30.0%	11 8.5%	73 56.1%	50 38.5%	−0.33 (1.35)
e. The stockpiling of medicines should be reduced and instead orders should be placed more frequently and in smaller package sizes.	12 9.2%	22 16.9%	32 24.6%	37 28.5%	27 20.8%	34 26.1%	64 49.3%	0.35 (1.24)
f. Employees should convey an environmentally conscious attitude (role model function).	36 27.7%	26 20.0%	17 13.1%	34 26.2%	17 13.1%	62 47.7%	51 39.3%	−0.23 (1.43)
g. In future, greater attention will be paid to the carbon footprint and environmentally friendly production of the materials used along supply chains.	33 25.4%	31 23.6%	41 31.5%	18 13.8%	7 5.4%	64 49.2%	19.2%	−0.50 (1.17)
